# Anal Canal Squamous Cell Carcinoma Following Low-Dose-Rate Prostate Brachytherapy: A Report of Two Cases and Literature Review

**DOI:** 10.7759/cureus.92602

**Published:** 2025-09-18

**Authors:** Naoto Shibuya, Takashi Fukagai, Yoshiki Tsunokawa, Kazuhiko Oshinomi, Masakazu Nagata, Masashi Morita, Masako Kato, Madoka Morota, Yoshinori Ito, Toshiko Yamochi

**Affiliations:** 1 Department of Urology, Showa Medical University School of Medicine, Tokyo, JPN; 2 Department of Radiology, Showa Medical University School of Medicine, Tokyo, JPN; 3 Department of Diagnostic Pathology, Showa Medical University School of Medicine, Tokyo, JPN

**Keywords:** anal canal squamous cell carcinoma, case report, low-dose-rate brachytherapy, prostate cancer, radiation-induced malignancy, secondary cancer

## Abstract

We report two cases of anal canal squamous cell carcinoma (SCC) that developed following low-dose-rate (LDR) brachytherapy for prostate cancer.

Case 1 involved a 73-year-old man who had undergone LDR brachytherapy (145 Gy) for prostate cancer (prostate-specific antigen (PSA) 8.3 ng/mL, Gleason score 3+3=6, T2N0M0) in December 2009. He remained disease-free for eight years and four months, but later presented with anal pain and an anal mass. Biopsy confirmed SCC. Based on CT findings demonstrating perineal invasion, the clinical stage was determined as cT4N1bM0, with right obturator lymph node metastasis.

Due to poor general condition, only diverting colostomy and external beam radiation therapy (EBRT, 70 Gy in 30 fractions) were performed. The patient died of aspiration pneumonia and sepsis on hospital day 94, in July 2018, eight years and seven months after the initial LDR treatment.

Case 2 was a 61-year-old man with prostate cancer (PSA 6.1 ng/mL, Gleason score 3+5=8, T1N0M0) treated with LDR brachytherapy (110 Gy) combined with EBRT (45 Gy in 25 fractions) in July 2006. He remained disease-free for eight years and seven months, after which he developed anal pain in February 2015. Endoscopic biopsy confirmed anal canal SCC (T1N1M0). Laparoscopic abdominoperineal resection (Miles’ operation) was performed, but local recurrence occurred one year later. Despite subsequent chemoradiation (5-fluorouracil plus mitomycin C) and systemic chemotherapy with XELOX (capecitabine plus oxaliplatin), the disease progressed, and he died in October 2016, 10 years and three months after the initial LDR treatment.

Anal canal SCC is a rare malignancy, and its development following prostate cancer radiotherapy is extremely uncommon. These two cases may represent radiation-induced secondary cancers, underscoring the importance of long-term surveillance for secondary malignancies in patients undergoing prostate brachytherapy.

## Introduction

Anal canal carcinoma is a rare malignancy, accounting for approximately 2.5% of all gastrointestinal tract cancers [1]. The age-adjusted incidence rate is about 1.5 to 2 per 100,000 population annually in Western countries, with a slightly higher prevalence among women than men [2]. In contrast, the incidence in Japan is even lower, estimated at approximately 0.3 per 100,000 population per year [3].

Histologically, the vast majority (85-90%) of anal canal cancers are squamous cell carcinomas (SCC), which are strongly associated with infection by high-risk (oncogenic) human papillomavirus (HPV) types, particularly HPV-16, the most common genotype linked to anal cancer development. Known risk factors include receptive anal intercourse, HIV infection, immunosuppression, and tobacco use [4].

Radiation therapy is an established curative treatment modality for localized prostate cancer, including low-dose-rate (LDR) brachytherapy and external beam radiation therapy (EBRT). However, radiation exposure to adjacent normal tissues may contribute to the development of second primary malignancies in long-term survivors. While several studies have documented an increased risk of bladder and rectal cancers following pelvic radiation for prostate cancer [5,6], anal canal carcinoma as a secondary malignancy is exceedingly rare, with only a few cases reported in the literature to date [7-9].

Between January 2005 and March 2021, we performed LDR brachytherapy for 1,511 patients with prostate cancer at our institution. During long-term follow-up, we encountered two cases of anal canal SCC that developed after prostate brachytherapy. Herein, we report two such cases and discuss the potential association between LDR brachytherapy and secondary anal canal carcinoma in the context of previous literature.

## Case presentation

Case 1

A 73-year-old man was diagnosed with low-risk prostate cancer (prostate-specific antigen (PSA) 8.3 ng/mL, Gleason score 3+3=6, cT1cN0M0) detected through PSA screening. Although active surveillance could have been considered, the patient expressed a preference for definitive treatment, and LDR brachytherapy was performed. He underwent LDR brachytherapy (145 Gy) in December 2009. Thereafter, his PSA level remained stable at <0.1 ng/mL without evidence of recurrence.

Follow-up was conducted every three to six months at our hospital for the first five years, until December 2014, after which PSA monitoring was continued at his primary care clinic. Anal pain developed in October 2017, but the patient did not seek immediate medical attention. In April 2018, he was finally referred to our gastroenterology department with anal pain and a protruding perianal mass. Physical examination revealed severe tenderness and a large tumor protruding through the anus. HPV testing was not performed in this case. The patient was negative for HIV, and there was no history of other sexually transmitted diseases. Serological testing for syphilis, including the Treponema pallidum hemagglutination assay (TPHA), was performed at admission and was negative. No underlying conditions or medications associated with immunosuppression were identified. He had a smoking history of approximately 15 cigarettes per day for 36 years (from age 20 to 56). Laboratory data revealed a mild elevation in C-reactive protein (CRP). Regarding tumor markers, the SCC antigen level was elevated (Table 1).

**Table 1:** Key laboratory findings at admission for Case 1

Parameter	Level	Reference
Hemoglobin	12	14.0-18.0g/dL
Total protein	5.2	6.7-8.4g/dL
Albumin	2.8	4.0-5.1g/dL
C-reactive protein (CRP)	1.6	0-0.2mg/dL
Carcinoembryonic antigen (CEA)	1.7	<5.0ng/mL
Squamous cell carcinoma antigen (SCC)	3.0	<1.5ng/mL

**Figure 1:**
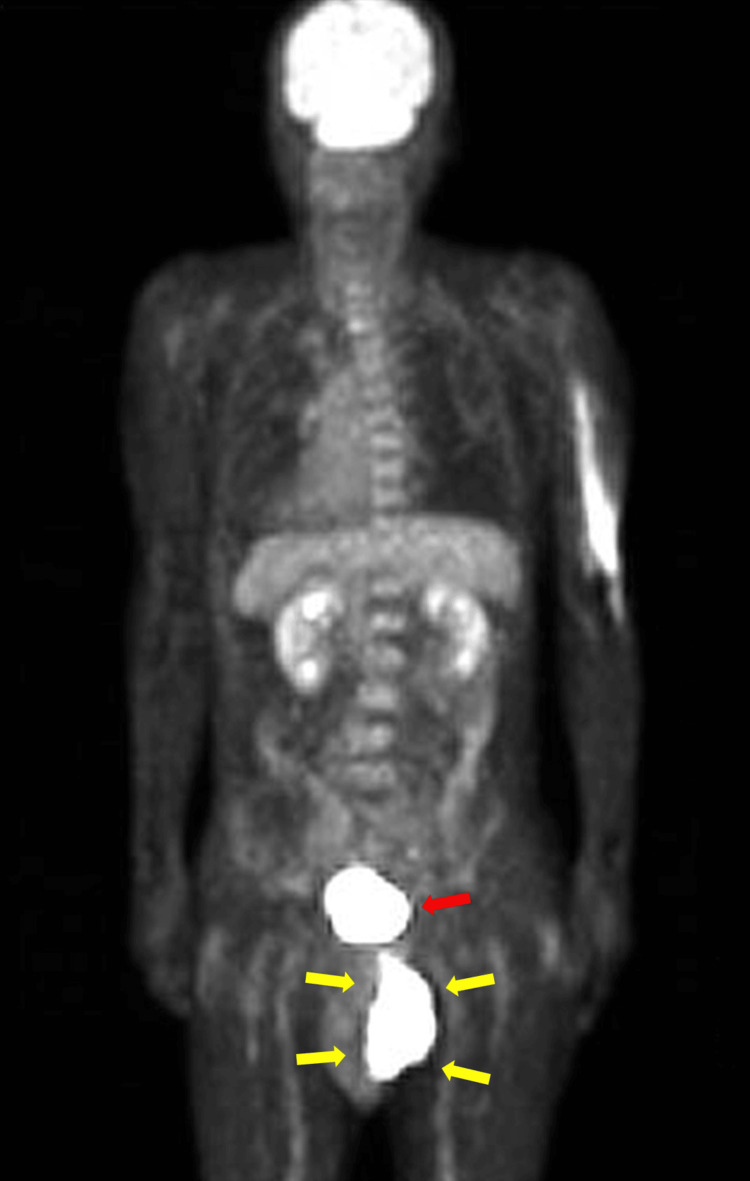
FDG-PET/CT scan of Case 1 (posterior-to-anterior view) Fluorodeoxyglucose positron emission tomography/computed tomography (FDG-PET/CT) scans showing a tumor in the anal canal (yellow arrows). Because the scan was performed in the prone position due to difficulty lying supine (the mass was protruding externally from the anus), the image orientation is posterior-to-anterior, resulting in a reversed appearance of the heart and other thoracic structures. The focal uptake in the right upper arm corresponds to extravasation of the radiotracer during injection. Physiological tracer accumulation in the urinary bladder is also visible (red arrow), located superior to the anal canal tumor.

**Figure 2:**
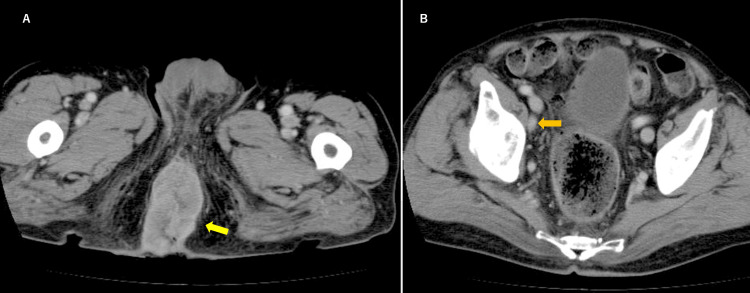
Contrast-enhanced CT images of Case 1 Contrast-enhanced CT showing anal canal cancer with suspected invasion into the perineum (A, yellow arrow) and metastasis to the right obturator lymph node (B, orange arrow).

Fluorodeoxyglucose positron emission tomography/computed tomography (FDG-PET/CT) scans revealed a tumor in the anal canal (Figure 1), and contrast-enhanced CT demonstrated suspected invasion into the perineum (Figure 2A, yellow arrow) and metastasis to the right obturator lymph node (Figure 2B, orange arrow).

**Figure 3:**
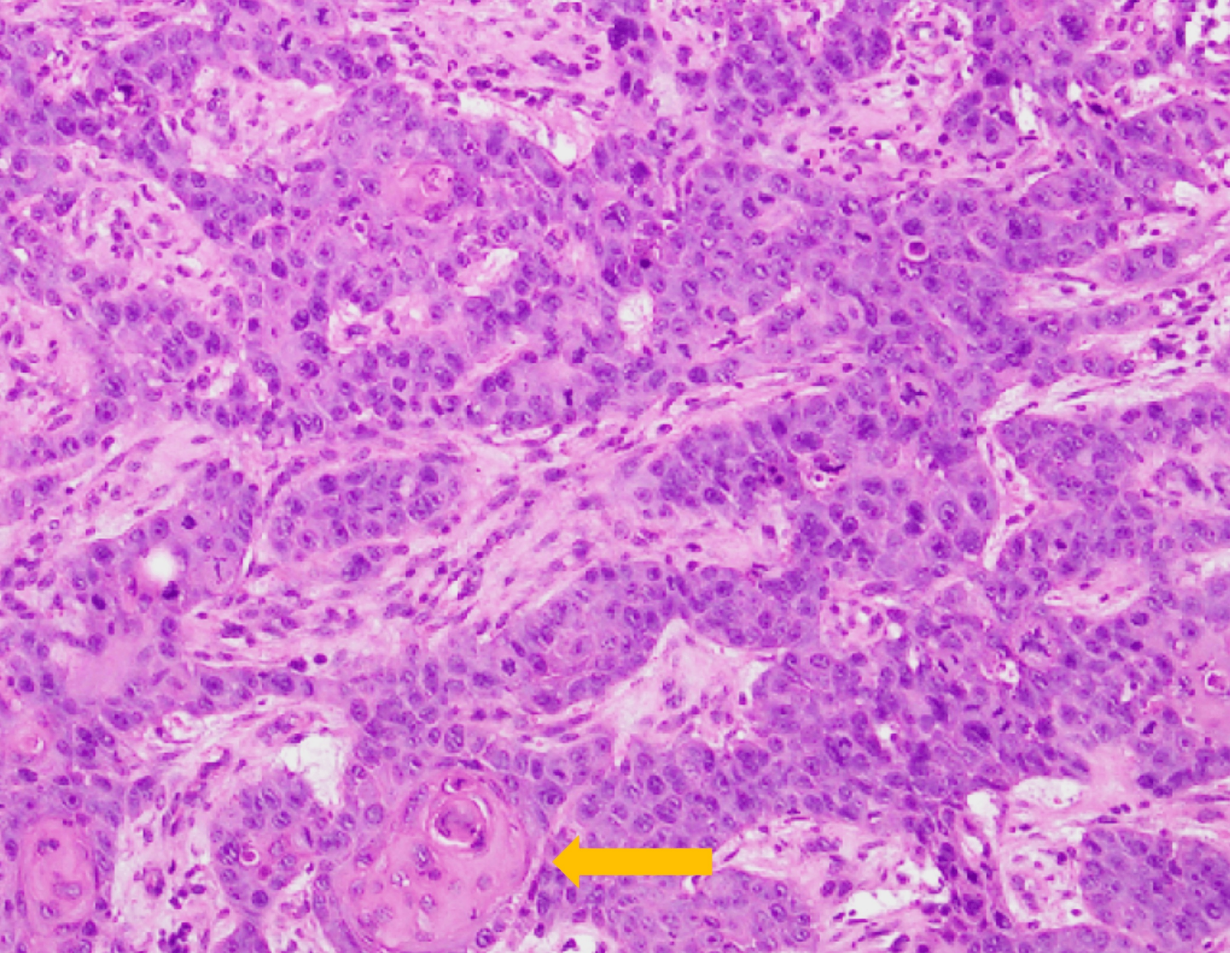
Histopathological findings of anal canal squamous cell carcinoma in Case 1 (hematoxylin and eosin stain) Atypical squamous cells with enlarged nuclei and hyperchromasia proliferating in a trabecular to nested pattern. The arrow indicates focal keratinization, characterized by eosinophilic material consistent with squamous differentiation.

No distant metastases were identified. Given the defecation difficulties caused by the tumor and his poor general condition, curative resection was deemed unfeasible. A sigmoid colostomy and tumor biopsy were performed. Histopathological examination of the biopsy specimen showed atypical squamous cells with enlarged nuclei and hyperchromasia forming trabecular and nested invasive patterns. Evidence of keratinization was also observed. Based on these findings, the tumor was diagnosed as a moderately differentiated SCC (Figure 3).

**Figure 4:**
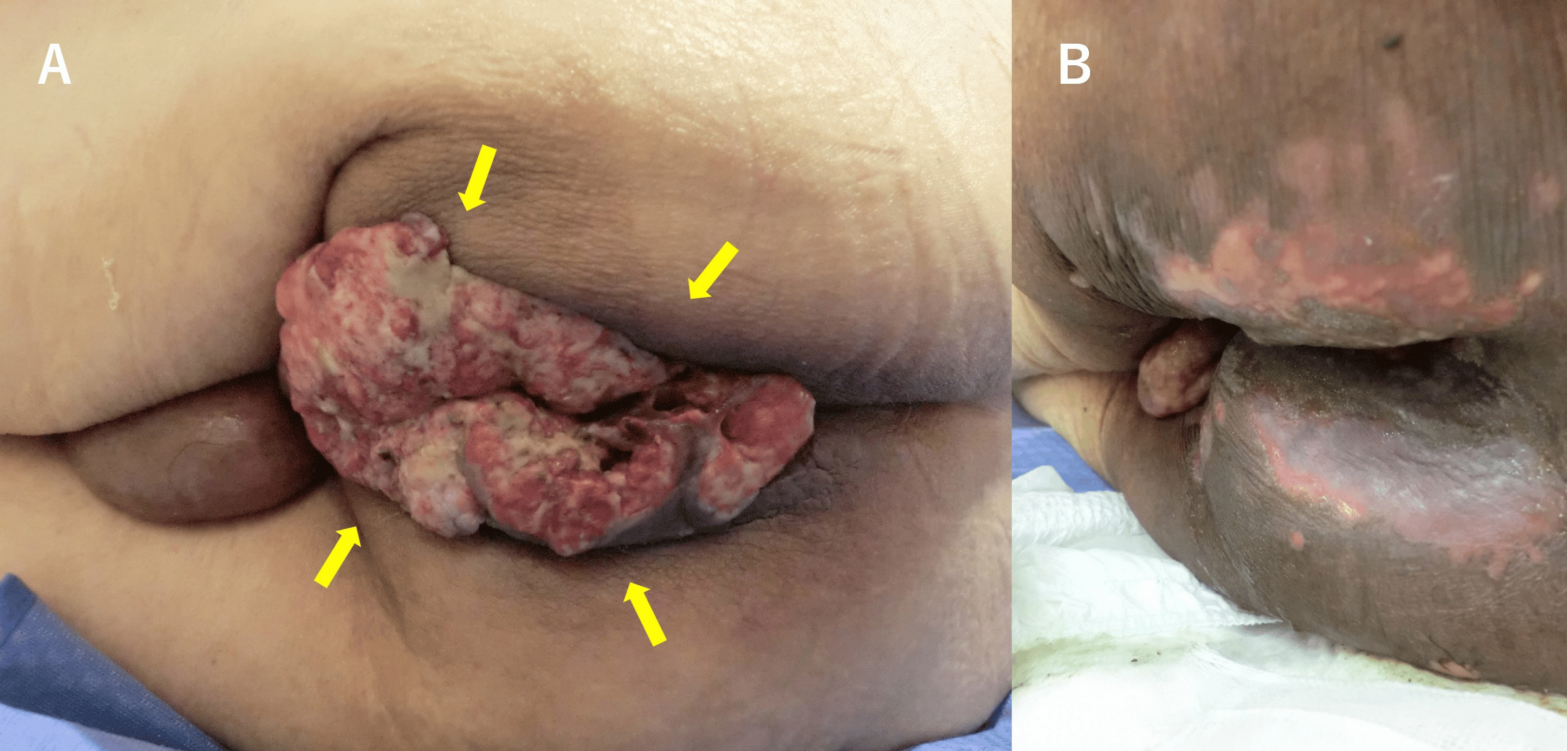
Clinical photographs of Case 1 before and after radiation therapy (A) Large, exophytic squamous cell carcinoma protruding from the anal canal before radiation therapy (yellow arrows). (B) Marked regression of the anal canal tumor following radiation therapy, with the mass almost completely resolved.

According to the American Joint Committee on Cancer (AJCC) eighth edition, the clinical stage was cT4N1bM0 anal canal SCC. Due to his compromised general condition, chemotherapy was not administered. Instead, he underwent colostomy and EBRT (70 Gy in 30 fractions). The radiation therapy achieved a marked local response, with significant regression of the protruding anal mass (Figure 4).

However, his general condition subsequently deteriorated, and he died in July 2018 at eight years and seven months after the initial prostate cancer treatment. The cause of death was aspiration pneumonia and sepsis, not cancer progression.

Case 2

The patient was a 61-year-old man diagnosed with high-risk, localized prostate cancer with a PSA of 6.1 ng/mL, Gleason score 3+5=8, and clinical stage cT1N0M0. In July 2006, he underwent LDR brachytherapy (110 Gy) combined with EBRT (45 Gy in 25 fractions) as definitive treatment. After the procedure, he was followed at our institution: for the first six years (until June 2012), he was monitored every three months with PSA testing, and thereafter every six months. His PSA decreased to 0.2 ng/mL and remained stable without evidence of recurrence. In February 2015, he developed anal pain and visited a local physician, who suspected anal canal carcinoma. He was then referred to our hospital’s Department of Gastroenterology for further evaluation and treatment. Laboratory data revealed elevated tumor markers, including SCC (5.2 ng/mL; normal <1.5 ng/mL) and CYFRA21-1 (3.7 ng/mL; normal <3.5 ng/mL), suggesting SCC (Table 2).

**Table 2:** Key laboratory findings at admission for Case 2

Parameter	Level	Reference
Hemoglobin	15.4	14.0-18.0g/dL
Total protein	7.9	6.7-8.4g/dL
Albumin	4	4.0-5.1g/dL
C-reactive protein (CRP)	0.4	0-0.2mg/dL
Carcinoembryonic antigen (CEA)	3.5	<5.0ng/mL
Squamous cell carcinoma antigen (SCC)	5.2	<1.5ng/mL
Cytokeratin fragment (CYFRA 21-1)	3.7	<3.5ng/mL

**Figure 5:**
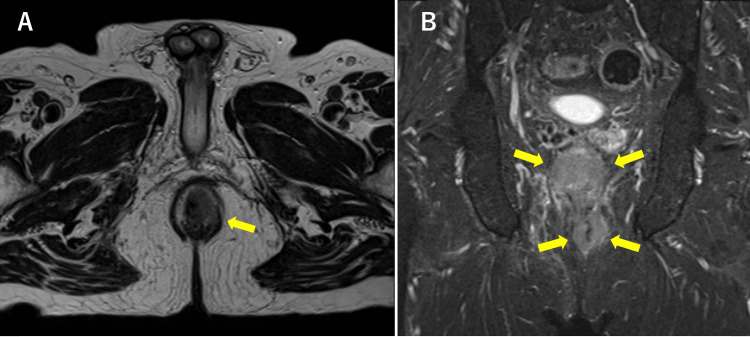
MRI axial and coronal views of Case 2 (A) Axial T2-weighted image showing a tumor lesion in the anal canal (yellow arrow), extending from the 12 o’clock to the 4 o’clock position and invading the perianal fatty tissue. (B) Coronal STIR image demonstrating abnormal signal intensity throughout the entire circumference of the lower rectum. The yellow arrows indicate the tumor extent encircling the anal canal and lower rectum. STIR: short tau inversion recovery

MRI demonstrated tumor invasion into the anterior anal sphincter and lower rectum (Figure 5A, yellow arrow). Coronal short tau inversion recovery (STIR) images revealed abnormal signal intensity throughout the circumference of the lower rectum (Figure 5B, yellow arrows).

**Figure 6:**
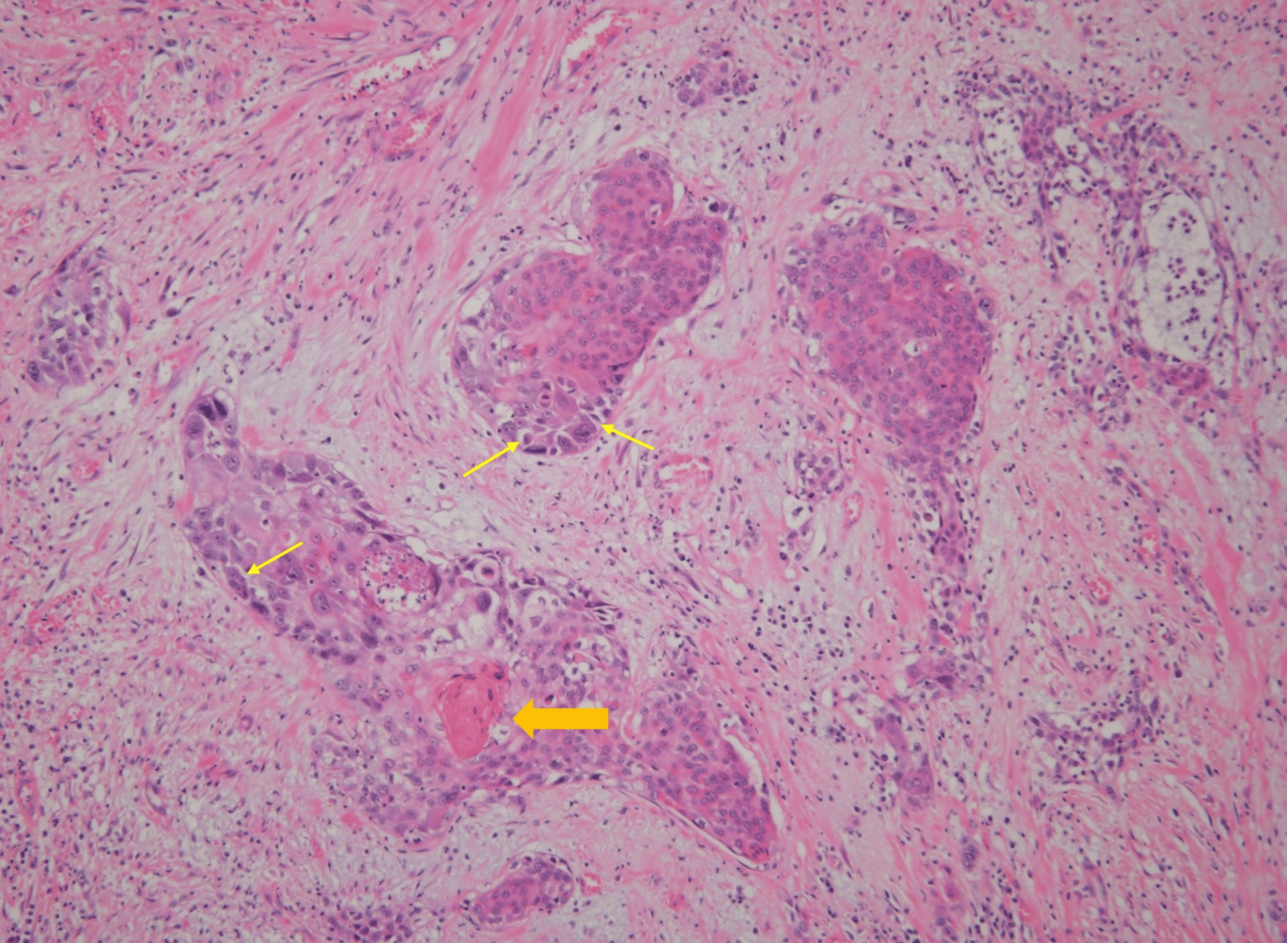
Histopathological findings of anal canal squamous cell carcinoma in Case 2 (hematoxylin and eosin stain) Tumor cells with nuclear atypia proliferating and infiltrating in a nested pattern, partially accompanied by keratinization (orange arrow), characterized by eosinophilic material consistent with squamous differentiation, and intercellular bridges (yellow arrows), representing desmosomal connections between adjacent squamous cells. These features confirm squamous differentiation.

No distant metastases were identified on CT. Colonoscopy with rectal biopsy confirmed SCC. HPV testing was also not performed in this case, and there was no history of other sexually transmitted diseases. Serological testing for syphilis (TPHA) was negative. No underlying conditions or medications associated with immunosuppression were identified. The patient had a smoking history of 20 cigarettes per day from age 20 to 50. The patient was admitted for treatment and underwent laparoscopic abdominoperineal resection (APR) (Miles’ operation) in March 2015. The resected specimen revealed a 70 × 70 × 30 mm mass located near the anal canal. Histopathologically, the tumor exhibited keratinization and intercellular bridges, consistent with a diagnosis of moderately differentiated SCC (Figure 6).

The tumor had invaded beyond the muscularis propria, with a positive deep surgical margin (pRM1) on the anterior side. Extensive lymphatic, vascular, and perineural invasion was observed. Two regional lymph node metastases were identified, and the cancer was classified as pT3N1aM0 according to the AJCC 8th edition [10]. Postoperatively, the patient was observed without adjuvant therapy. However, local recurrence was confirmed by CT in January 2016. He subsequently received EBRT (30 Gy in 10 fractions) combined with chemotherapy using 5-fluorouracil and mitomycin C; however, the response was limited. Because renal dysfunction precluded the use of cisplatin, XELOX (capecitabine plus oxaliplatin), commonly used for colorectal cancer, was selected after discussion between the oncology team and the patient. Despite this treatment, the disease progressed, and he died in October 2016. The survival time was approximately 10 years and three months after the initial LDR brachytherapy, with a disease-specific cause of death attributable to anal canal SCC.

DVH analysis of the anal canal in Cases 1 and 2 is summarized in Table 3.

**Table 3:** Anal canal DVH parameters for two patients treated with prostate brachytherapy * EBRT was delivered with an older treatment system, and the original DVH data were not digitally preserved; therefore, only the brachytherapy component could be analyzed. DVH: dose-volume histogram; BT: brachytherapy; EBRT: external beam radiotherapy; Vx%: volume (cc) receiving ≥x% of the prescription dose; D2cc: minimum dose to the most irradiated 2 cc of the anal canal; D0.1cc: minimum dose to the most irradiated 0.1 cc; mean dose: average dose to the entire anal canal

Parameter	Case 1 (BT 145 Gy)	Case 2 (BT 110 Gy + EBRT 45 Gy*)
V100% (cc, %)	0.00 (0%)	0.00 (0%)
V50% (cc, %)	0.00 (0%)	0.85 (6.99%)
V30% (cc, %)	0.78 (6.88%)	3.06 (24.43%)
D2cc (Gy, %)	36.01 (22.5%)	41.02 (37.3%)
D0.1cc (Gy, %)	65.45 (40.9%)	80.17 (72.9%)
Mean Dose (Gy, %)	24.60 (15.4%)	23.70 (21.6%)

In Case 1, the anal canal received a D2cc of 36.0 Gy (22.5% of the prescription dose) and a mean dose of 24.6 Gy (15.4%). In Case 2, the anal canal received a D2cc of 41.0 Gy (37.3%) and a mean dose of 23.7 Gy (21.6%) from the brachytherapy component (110 Gy). Although EBRT was delivered at 45 Gy in 25 fractions, the original dose-volume histogram (DVH) data were not digitally preserved due to the older treatment system, and therefore, only the brachytherapy component could be analyzed.

**Figure 7:**
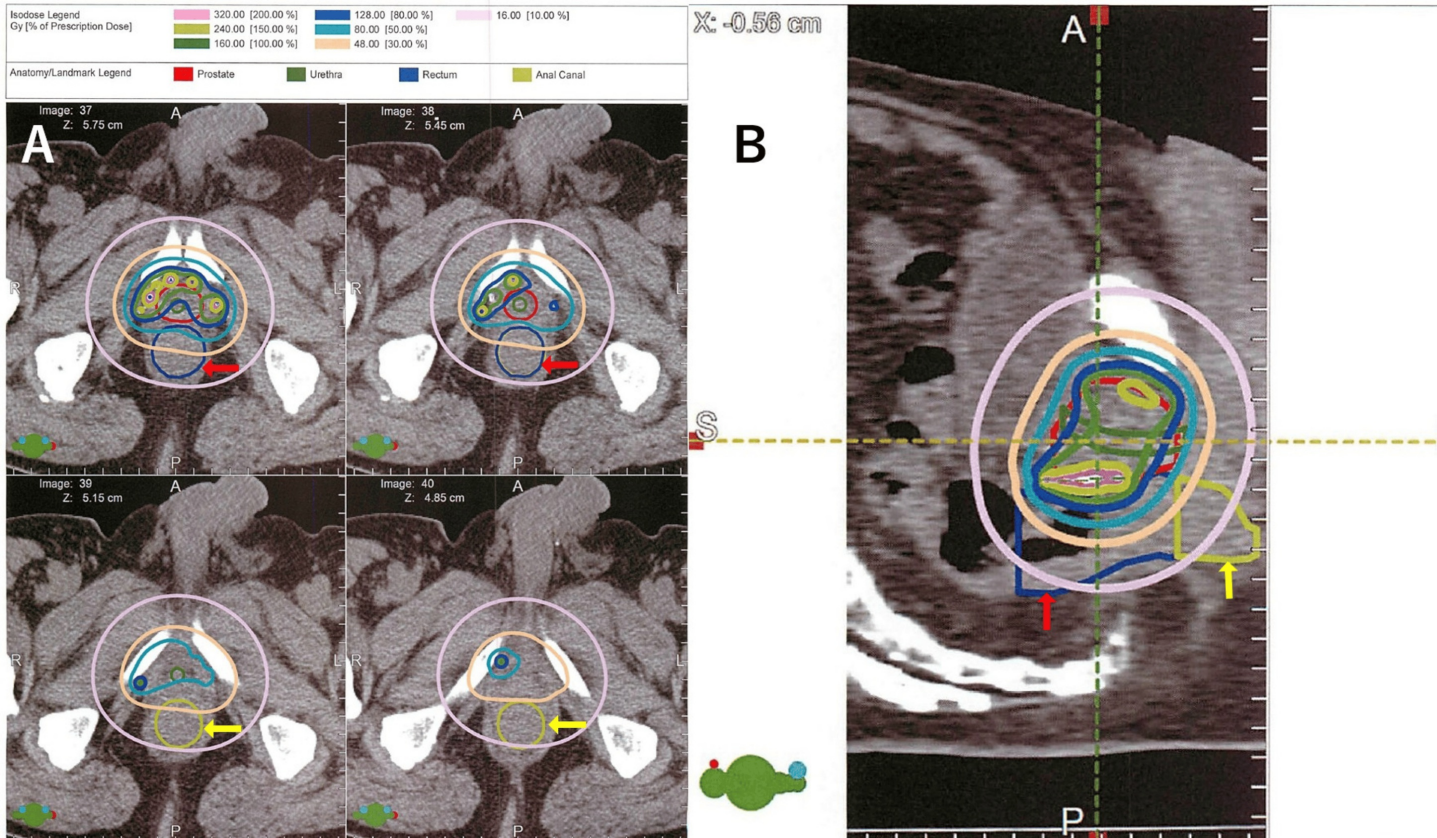
Post-implant dosimetry of Case 1 (A) Axial post-implant CT images showing the contoured rectum (red arrows) and anal canal (yellow arrows). (B) Sagittal reconstruction with isodose lines. The anal canal contour (yellow) demonstrates that although high-dose regions did not extend into the canal, low-to-moderate doses were delivered.

**Figure 8:**
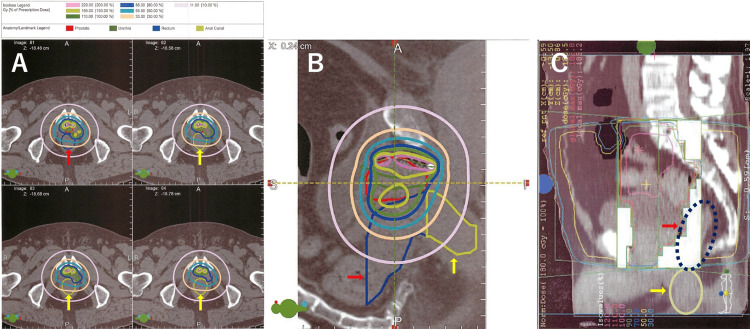
Post-implant and external beam radiotherapy plans of Case 2 (A) Axial CT slices showing the contoured rectum (red arrows) and anal canal (yellow arrows). (B) Sagittal reconstruction with isodose distribution. (C) Archived DVH printout of the EBRT component (45 Gy). Although digital DVH data were not preserved due to the older treatment system, this figure demonstrates the delivered external beam plan in relation to the anal canal region. DVH: dose-volume histogram; EBRT: external beam radiotherapy

Representative post-implant dosimetry plans with anal canal and rectal contours are shown in Figure 7 (Case 1, axial and sagittal views) and Figure 8 (Case 2, axial and sagittal views, including archived EBRT DVH printout).

These figures illustrate seed distribution near the prostatic apex and demonstrate that, while high-dose regions did not extend into the anal canal, both cases received non-negligible low-to-moderate radiation exposure.

## Discussion

Radiation-induced malignancies are believed to arise from the accumulation of DNA damage and genomic instability caused by ionizing radiation, particularly in normal tissues located within or adjacent to the irradiated field. These changes may lead to mutations in tumor suppressor genes, impaired DNA repair mechanisms, and a pro-inflammatory microenvironment, ultimately promoting carcinogenesis over a latency period of several years to decades [11,12]. While direct radiation carcinogenesis has long been accepted, there is also evidence that irradiation of the prostate might contribute to carcinogenesis outside the irradiated area through radiation scatter and radiation-induced genetic alterations without direct exposure, possibly mediated by increased reactive oxygen species and changes in gene expression in what has been termed the “bystander effect” [13,14]. Previous studies have reported an increased risk of secondary cancers such as bladder and rectal carcinomas following pelvic radiation therapy for prostate cancer [5,15]. In contrast, radiation-associated anal canal SCC is exceedingly rare and has not been clearly documented in large-scale epidemiological studies to date. Although differential diagnosis with rectal cancer could be considered in our cases, several findings support the anal canal as the primary site: in Case 1, imaging demonstrated a tumor protruding externally from the anal canal with marked response to radiotherapy, while in Case 2, colonoscopy and pathological examination of the resected specimen confirmed localization to the anal canal and a squamous histology. Moreover, SCC of the rectum itself is extremely rare. Taken together, these findings strongly indicate that both tumors originated in the anal canal rather than the rectum. No cases of anal canal SCC following LDR brachytherapy have been included in large-scale reviews; however, only three individual cases have been previously reported, and the present two cases represent the fourth and fifth worldwide [7-9]. A summary of these five cases is presented in Table 4.

**Table 4:** List of publications reporting anal canal cancer following brachytherapy for prostate cancer Staging: Anal canal carcinoma (ACC) staging was performed according to the American Joint Committee on Cancer (AJCC) eighth edition, when available. PSA units are reported in ng/mL. Gleason score is presented as primary + secondary = total. ^†^ Age at prostate cancer (PCa) diagnosis was inferred from the age at ACC diagnosis minus the reported interval, when not explicitly stated in the source. PSA: prostate-specific antigen; APR: abdominoperineal resection; BT: brachytherapy; LDR: low-dose-rate; CRT: chemoradiotherapy; EBRT: external beam radiotherapy; NR: not reported; RS-RARP: Retzius-sparing robot-assisted radical prostatectomy; SCC: squamous cell carcinoma; HPV: human papillomavirus

Author (Year)	Age at PCa Dx (y)	PSA (ng/mL)	Gleason	PCa Clinical Stage	BT Dose (Gy)	EBRT (Gy)	Age at ACC Dx (y)	Interval From BT to ACC	ACC Histology	ACC Stage	HPV/p16	Treatment for ACC	Outcome After ACC
Apinorasethkul et al. (2016) [7]	66^†^	NR	NR	NR	145	No	76	10 years	SCC	cT3N0M0	NR	Chemo/RT (proton ± electrons)	NR
Hirose et al. (2020) [8]	NR	6.58	3+3=6	cT1cN0M0	145	No	70s	Eight years	SCC	pT2N1aM0	NR	APR (laparoscopic) + CRT	Died of disease (four years and six months)
Yanase et al. (2025) [9]	67^†^	NR	NR	cT2aN0M0	NR	NR	76	Nine years	SCC	pT3N0M0	NR	Robot-assisted APR + additional RS-RARP	No recurrence at last follow-up
Case 1 (current)	73	8.3	3+3=6	cT1cN0M0	145	No	81	Eight years and four months	SCC	cT4N1bM0	Not performed	Stoma + RT (70 Gy)	Died (three months)
Case 2 (current)	61	6.1	3+5=8	cT1cN0M0	110	45	70	Eight years and seven months	SCC	pT3N1aM0	Not performed	APR (laparoscopic) + CRT + chemo	Died (one year and seven months)

As noted above, in Japan, the annual incidence rate of anal canal cancer is approximately 0.3 per 100,000 population [3]. Therefore, in our institution, the occurrence of two such cases among approximately 1,500 patients treated with LDR brachytherapy for prostate cancer appears somewhat higher than expected by chance alone. However, it should be noted that the incidence of anal canal cancer in Japan is strongly influenced by the relatively low prevalence of high-risk HPV infection [16]. In our two cases, HPV/p16 testing was not performed, and although both patients were HIV-negative with no immunosuppressive conditions, lifestyle-related risk factors such as smoking were present, and sexual health histories were not available. Therefore, HPV-associated carcinogenesis cannot be excluded, and the observed incidence should be interpreted with caution. Although it is difficult to definitively conclude that these cases represent radiation-induced malignancies, in all reported cases, including our own, the tumors developed within the low-dose radiation field of LDR brachytherapy, with a latency period of 8 to 10 years after treatment, and all were histologically SCCs. Notably, keratinization and intercellular bridges were observed in our pathological specimens, which are well-recognized histological hallmarks of squamous differentiation, further supporting the diagnosis of SCC rather than other histological subtypes. To further evaluate this possibility, we summarized the applicability of Cahan’s criteria for radiation-induced malignancy across all five cases reported to date (Table 5) [17].

**Table 5:** Summary of Cahan’s criteria in five cases of anal canal squamous cell carcinoma following prostate brachytherapy SCC: squamous cell carcinoma; NR: not reported; HPV: human papillomavirus; TPHA: Treponema pallidum hemagglutination assay

Cahan’s Criteria	Case 1 (Current)	Case 2 (Current)	Apinorasethkul et al., 2016	Hirose et al., 2020	Yanase et al., 2025
1. Different histology from the primary tumor	Yes (prostate adenocarcinoma → anal canal SCC)	Yes	Yes	Yes	Yes
2. Tumor arises within the prior radiation field	Yes (anal canal adjacent to prostate brachytherapy seeds)	Yes	Yes	Yes	Yes
3. Sufficient latency (>5 years)	Yes (eight years and four months)	Yes (eight years and seven months)	Yes (10 years)	Yes (eight years)	Yes (nine years)
4. Exclusion of other causes	Partially (HPV not tested; HIV-, TPHA-; smoker)	Partially (HPV not tested; HIV-, TPHA-; smoker)	NR (HPV status not reported)	NR	NR

As shown, criteria 1-3 were fulfilled in all cases, whereas criterion 4 (exclusion of other etiologies) could only be partially satisfied because HPV/p16 status was not assessed in our two cases and not reported in the others. Therefore, while these tumors may represent radiation-associated anal canal cancers, the contribution of HPV-related carcinogenesis cannot be excluded, and caution is warranted in attributing causality.

Interestingly, no cases of anal canal cancer have been reported following EBRT, whereas all reported cases, including ours, occurred after LDR brachytherapy.

In our cases, the rectal dose was not particularly high, with a post-plan rectal V100 of 0.26 cc in Case 1 (prescribed dose 145 Gy) and 0.01 cc in Case 2 (prescribed dose 110 Gy). However, DVH analysis of the anal canal (Table 3) and representative dosimetry plans with anal canal contours (Figures 7-8) revealed that, while high-dose regions did not extend into the anal canal, low-to-moderate radiation exposure was delivered to this region. These findings suggest that the anal canal, particularly near the prostatic apex, may receive clinically relevant radiation even when rectal doses are within acceptable limits. As a result, compared with EBRT, the upper anal canal adjacent to the prostatic apex is subjected to a low but continuous radiation exposure over a long period of time. Such chronic exposure may cause long-term DNA damage and local chronic inflammation, potentially contributing to tumorigenesis.

Another notable finding is that four of the five reported cases, including the present ones, occurred in Japanese patients. Anal canal cancer is generally rare in Japan, and unlike in Western countries, where SCC predominates, Japanese cases have been reported to show a relatively higher proportion of adenocarcinoma [16]. Given this background, the occurrence of exclusively SCC histology in all reported radiation-associated cases raises the possibility that there may be racial or regional differences in susceptibility to radiation-induced anal canal cancer. Although the biological basis for this observation is unknown, further epidemiological and molecular studies are warranted to explore potential genetic or environmental factors contributing to this apparent predisposition.

With regard to treatment, chemoradiotherapy (CRT) with 5-fluorouracil and mitomycin C in combination with external beam radiotherapy is considered the standard of care for anal canal SCC [18]. However, in patients with a history of prostate brachytherapy, there is concern that additional high-dose irradiation to the pelvic region may result in excessive cumulative dose to previously irradiated tissues, leading many clinicians to opt for surgical resection, such as APR, as the primary treatment. Notably, one previously reported case was successfully treated with proton beam therapy after careful assessment of the prior dose distribution, suggesting that re-irradiation with advanced radiation modalities may be feasible in selected patients. These cases highlight the importance of individualized treatment planning, taking into account prior radiation fields, cumulative dose constraints, and the patient’s overall condition. These experiences underscore the importance of individualized treatment strategies for this rare condition.

In the present study, including our two cases, only five cases have been analyzed; therefore, a definitive causal relationship between prostate brachytherapy and the development of anal canal cancer cannot be established. Given that anal canal cancer is a rare malignancy with inherently low case numbers, nationwide and international multicenter collaborations are warranted. Statistical evaluation of the relationship between brachytherapy and the occurrence of anal canal cancer, as well as detailed investigations into the association between radiation dose to the anal canal and molecular biological changes, will be necessary in the future.

## Conclusions

We reported two cases of anal canal SCC developing after LDR brachytherapy for prostate cancer. In patients with a history of prostate brachytherapy, long-term surveillance is essential, and the onset of lower gastrointestinal symptoms should raise suspicion not only for cancer recurrence and radiation-related adverse events such as proctitis, but also for secondary malignancy. When such symptoms occur, clinicians should carefully consider the need for digital rectal examination and/or endoscopic evaluation. Further accumulation of cases and detailed analyses of dose-volume parameters are warranted to clarify the risk of anal canal cancer after brachytherapy and to develop effective preventive strategies.
